# Neuralgia

**Published:** 1900-03-15

**Authors:** 


Neuralgia.—Equal parts of benzoin and oil of peppermint rubbed on the affected part, or sprinkled on a cloth wrung out of hot water, in many cases acts like a charm—*Pacific Medical Gazette.*

